# Supplementary material for: Physics to system-level modeling of silicon-organic-hybrid nanophotonic devices
Source: Sci Rep. 2024 May 23;14:11751. doi: 10.1038/s41598-024-61618-x (PMC11116435; doi:10.1038/s41598-024-61618-x)
Supplement: Supplementary file 1 — Supplementary Information. [file 41598_2024_61618_MOESM1_ESM.docx]

**Supplementary Information****: *Design Methodology for Silicon Organic Hybrid Modulators: From Physics to System-level Modeling***

**Maryam Moridsadat^1^, Marcus Tamura^1^, Lukas Chrostowski^2^, Sudip Shekhar^2^,
Bhavin J. Shastri^1^**

^1^ Department of Physics, Engineering Physics & Astronomy, Queen’s University, Kingston, ON K7L 3N6, Canada

^2^ Department of Electrical and Computer Engineering, University of British Columbia, Vancouver, BC V6T 1Z4, Canada

Supplementary Fig. **1** depicts the SOH MRR coupling coefficient vs. wavelength.

| 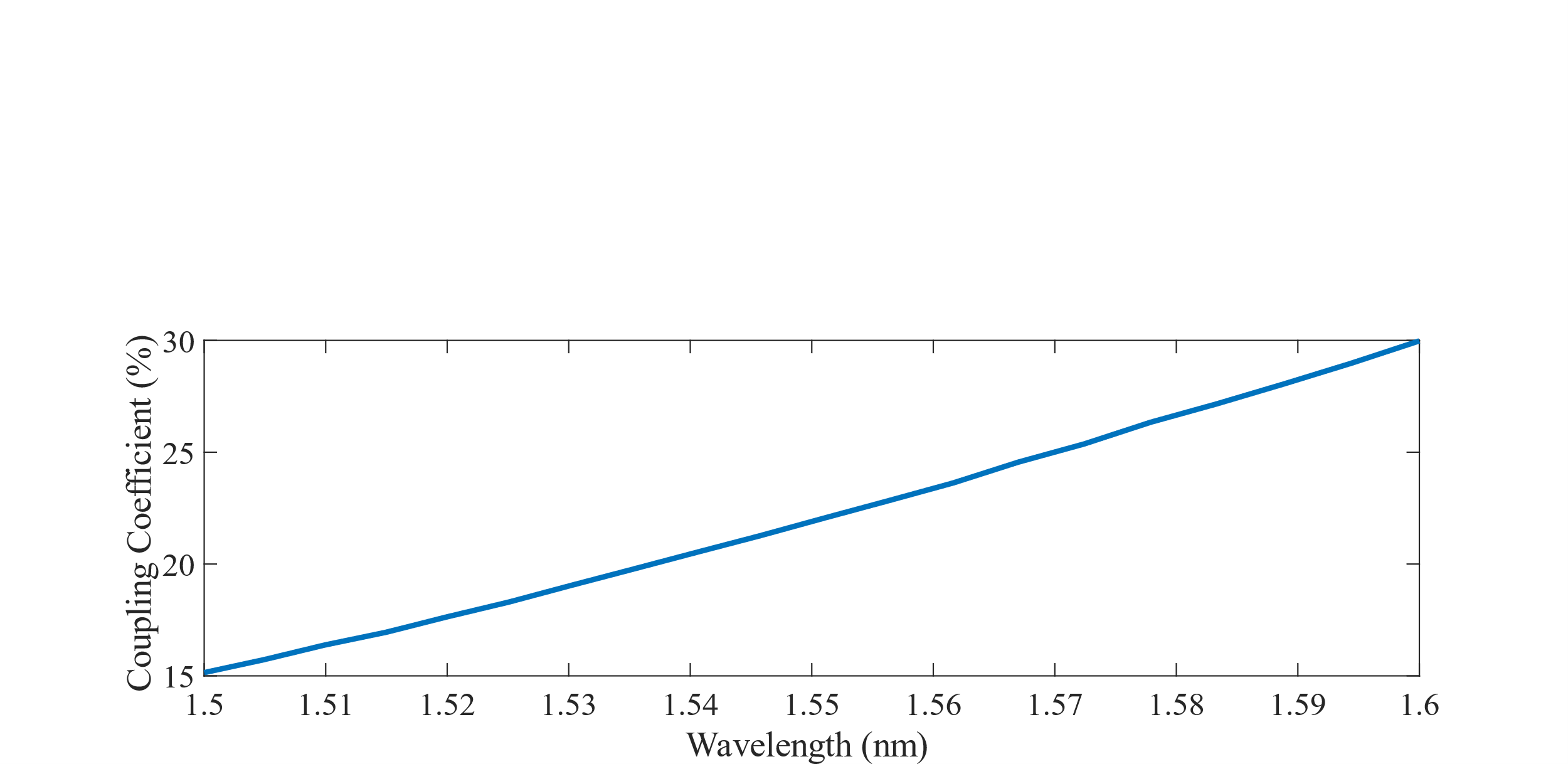 |
| --- |
| **Supplementary Fig. 1.**The coupling coefficient power of the fundamental TE mode vs. wavelength for the studied SOH MRM. |

Supplementary Fig. **2** a and b depict the *x*-component of the optical E-field of the straight and bent waveguides of the MRM at the wavelength of 1550 nm. Supplementary Figure S2 also shows the real part of the refractive index of the straight and bent waveguides of the MRM vs. wavelength.

| 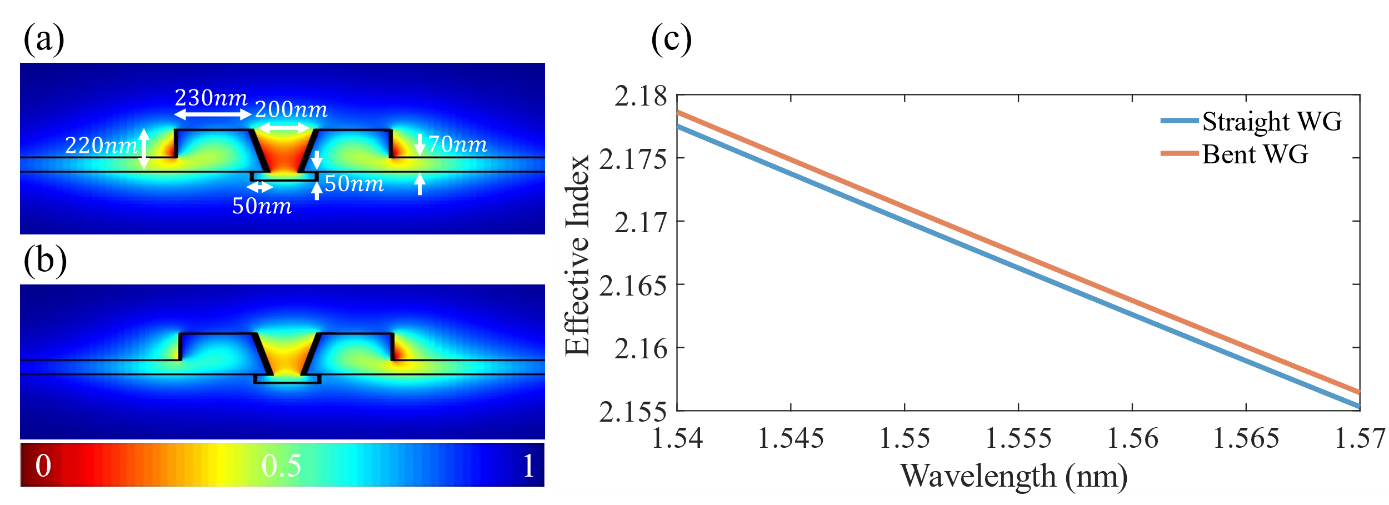 |  |
| --- | --- |
| **Supplementary Fig. 2**. The MRR’s waveguide optical characteristics. **(a)** The x-component optical E-field ($E_{x}$) profile of straight waveguide and **(b)** bent waveguide, both at 1550nm. The E-field is normalized to its maximum intensity. **(c)** The real part of the effective index of straight and bent waveguides vs. wavelength. | |

Supplementary Fig. 3 illustrates the building-block based simulation platform of the SOH MRM in Lumerical INTERCONNECT, including all the MRR subcomponents elements.

| 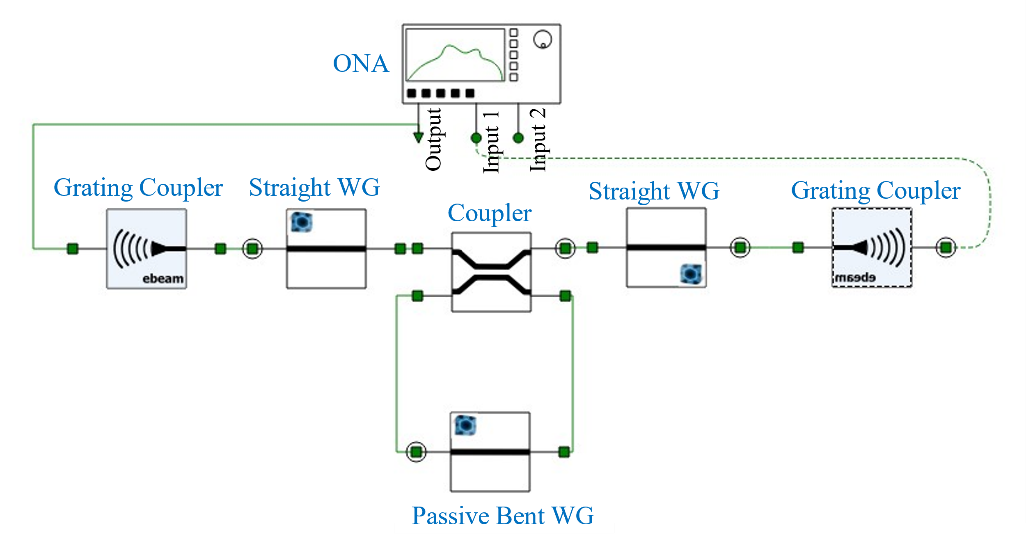 |
| --- |
| **Supplementary Fig. 3** Passive simulation platform of the SOH MRM in Lumerical INTERCONNECT by assembling all the MRR subcomponents elements and loading the correlated physics-based simulations results. |

Supplementary Fig. **4** shows the electrical E-field intensity in and around the MRM’s waveguide at various voltages, including V=1,2, and 3 V.

| 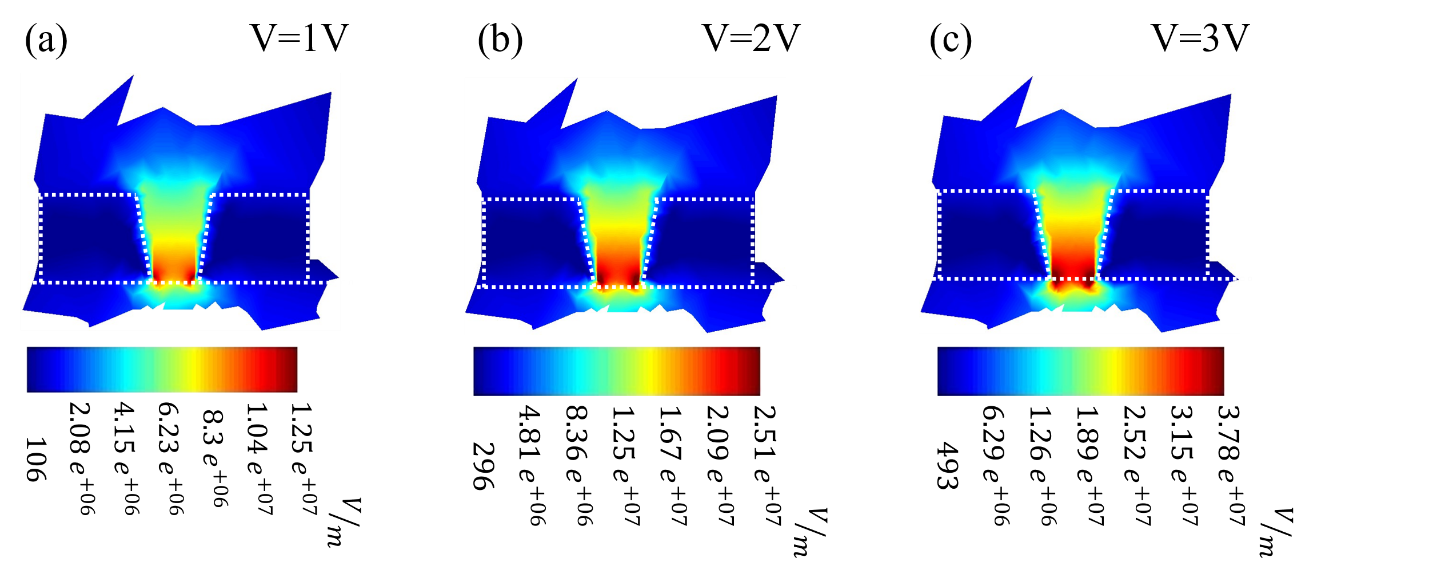 |
| --- |
| **Supplementary Fig. 4**. electrical E-field at various voltages for the studied SOH MRM. **(a)** 1 V, **(b)** 2 V, and **(c)** 3 V. |

Supplementary Fig.5 a depicts the simulation of the inserted electrical E-field profile from Lumerical CHARGE to the MODE FDE at 1V. The effective index change as a function of voltage at the wavelength of 1552.41 nm has also been depicted in Supplementary Fig**.5** b.

| 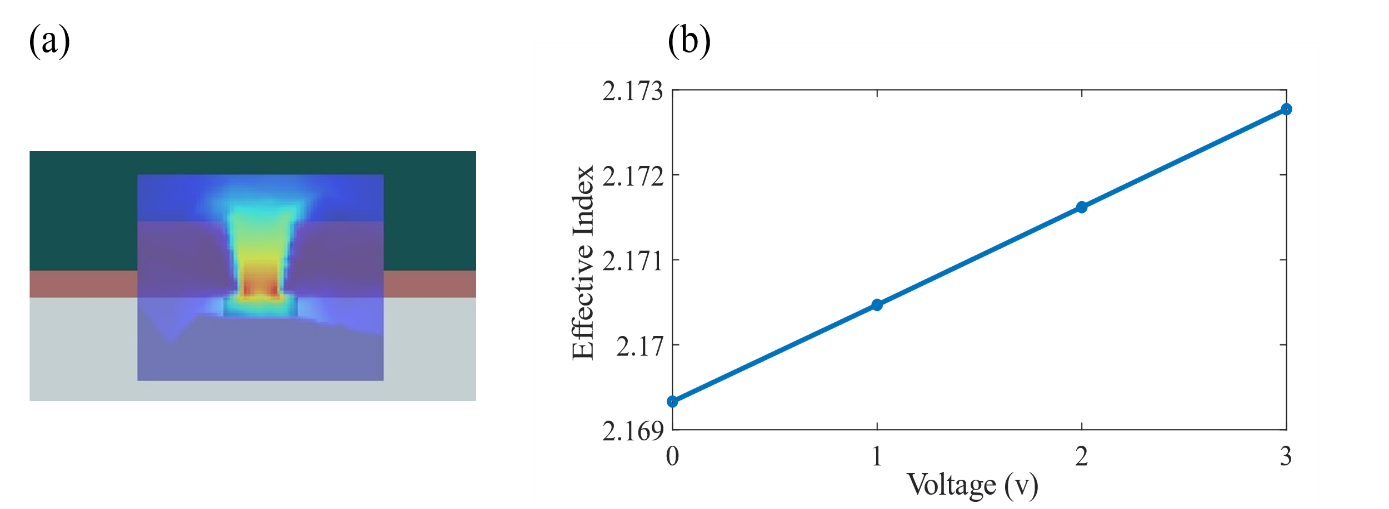 |
| --- |
| **Supplementary Fig.5.** The electrical E-field distribution profile, and effective index vs. voltage for the MRR. **(a)** The electrical E-field distribution profile from Lumerical CHARGE inserted in MODE simulation region at V=1. **(b)** The change of effective index as a function of voltage at 1552.41 nm. |

Supplementary Fig. **6** illustrates the electro-optical simulation platform of the SOH MRM, including both optical and electrical stimuli, in Lumerical INTERCONNECT.

| 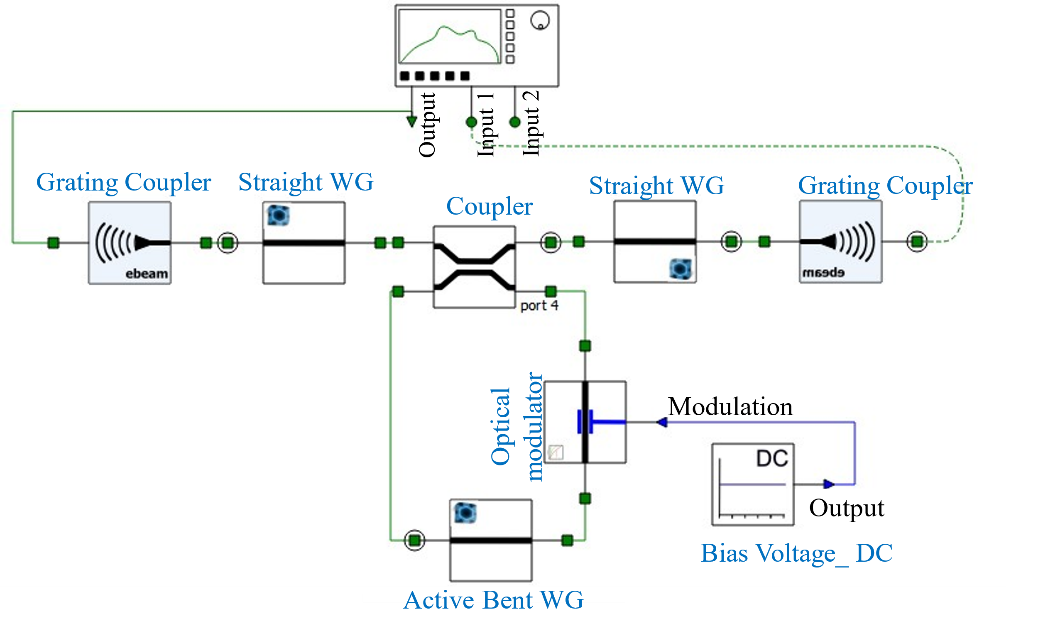 |
| --- |
| **Supplementary Fig. 6** The electro-optical simulation methodology platform of the SOH MRM in the Lumerical INTERCONNECT. |

Supplementary Fig. 7 a, b, and c show the physics-level simulation of the SOH MZM’s waveguide, including the optical E-field, electrical E-field, and CHARGE solver simulation results incorporated in MODE solver to realize the effective refractive index at 0.9 V ($V_{\pi}$).

| 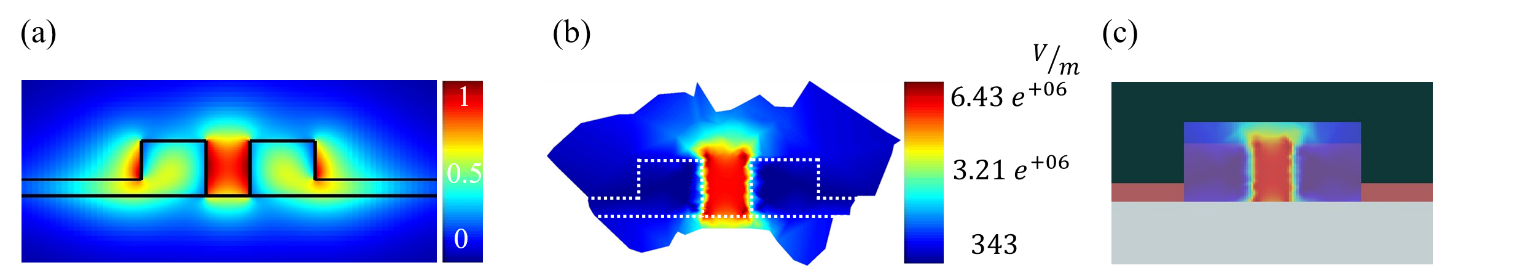 |
| --- |
| **Supplementary Fig. 7**. Physics-level simulation of MZM’s waveguide. **(a)** The optical and **(b)** electrical E-field profile simulated in MODE (at 1550 nm wavelength) and CHARGE (at v=0.9 V), respectively. **(c)** Charge-results incorporated in Mode FDE solver to achieve the effective index at 0.9 V. |

Supplementary Fig. **8** shows the electro-optical co-simulation platform of the SOH MZM in Lumerical INTERCONNECT.

| 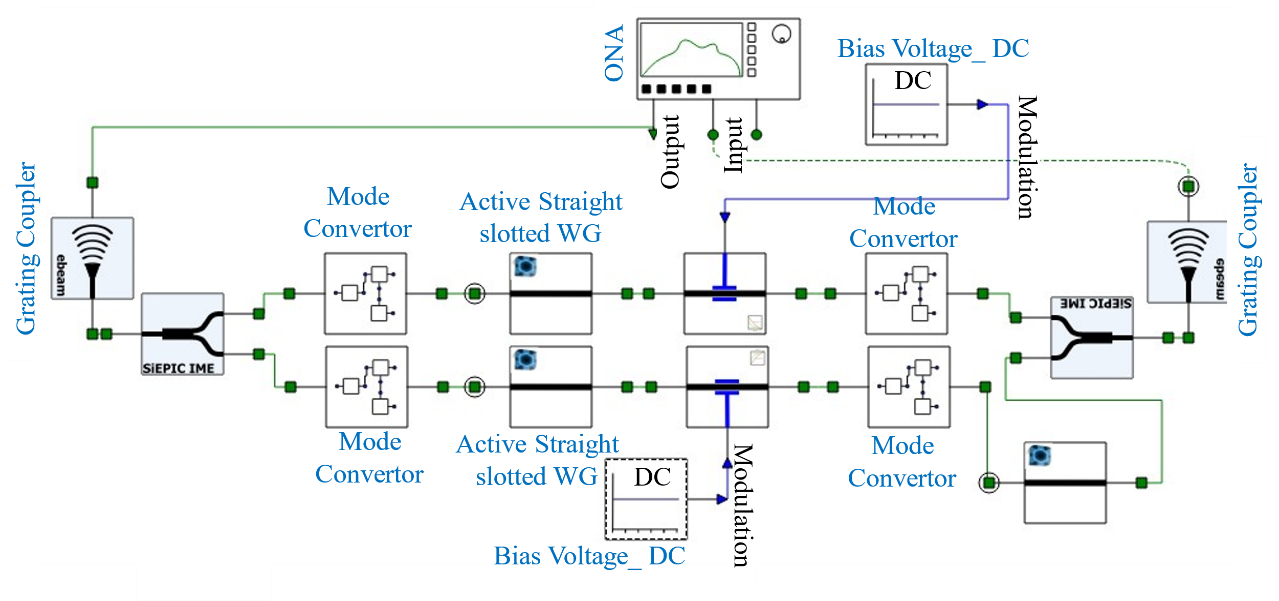 |
| --- |
| **Supplementary Fig. 8** The circuit-level electro-optical simulation platform of the SOH MZI in the Lumerical INTERCONNECT |
